# DNAJB4 suppresses breast cancer progression and promotes tumor immunity by regulating the Hippo signaling pathway

**DOI:** 10.1007/s12672-023-00762-8

**Published:** 2023-08-07

**Authors:** Yanru Chen, Jingjia Li, Lulan Pu, Jinghua Hu, Lingyu Fang, Fangfang Zhou, Hongying Zhang, Yi Yang, Xinxin Rong, Shishan Deng, Lingmi Hou

**Affiliations:** 1 Academician (Expert) Workstation, Medical Imaging Key Laboratory of Sichuan Province, Biological Targeting Laboratory of Breast Cancer, Department of Breast and Thyroid Surgery, Affiliated Hospital of North Sichuan Medical College, Nanchong, Sichuan China; 2 Medical Imaging Key Laboratory of Sichuan Province, Institute of Basic Medicine and Forensic Medicine, North Sichuan Medical College, Nanchong, Sichuan China; 3 The Fifth People’s Hospital of Nanchong City, Nanchong, Sichuan China

**Keywords:** Breast cancer, DNAJB4, Migration, Proliferation, Hippo pathway, Tumor immunity

## Abstract

**Purpose:**

Breast cancer is the most common cancer worldwide. Low DNAJB4 expression levels are strongly correlated with poor prognosis in breast cancer patients. However, the molecular mechanism by which DNAJB4 regulates breast cancer progression is unclear.

**Methods:**

The expression of DNAJB4 was validated in human breast cancer tissues, normal human breast tissues, and breast cancer cell lines. CCK-8, colony-forming, and wound healing assays were used to assess the biological effect of DNAJB4 overexpression on cell proliferation and migration in MCF-7 cell lines. Bioinformatic analysis was used to identify the DNAJB4 related pathways in breast cancer. Epithelial-mesenchymal transition (EMT)-related biomarkers and Hippo pathway components were quantified by Western blots. Luciferase and Western blot assays were used to validate which miRNA regulates DNAJB4. In addition, the effects of DNAJB4 on in vivo tumor growth were assessed in xenograft models.

**Results:**

DNAJB4 was expressed at low levels in human breast cancer tissues and breast cancer cell lines and correlated with poor prognosis. DNAJB4 overexpression significantly inhibited cell proliferation and migration in vitro by activating the Hippo pathway. The dual-luciferase assay showed that hsa-miR-183-5p targeted DNAJB4. Moreover, the effects of DNAJB4 could be reversed by miR-183-5p. In addition, the expression of DNAJB4 was strongly correlated with immune infiltration levels. Notably, DNAJB4 overexpression markedly enhanced CD4 + and CD8 + T cells and reduced PD-L1 levels in 4T1 tumors via the Hippo pathway, which retarded tumor growth in a subcutaneous xenograft tumor mouse model of 4T1 cells.

**Conclusions:**

The present study demonstrated that DNAJB4 overexpression inhibited the malignant biological behavior of breast cancer by regulating the Hippo pathway and tumor immunosuppressive environment.

## Introduction

Globally, breast cancer ranks fifth in terms of cancer deaths [1]. The global incidence of breast cancer has now passed lung cancer as the number one cause of women cancer mortality in 2020 [2]. Based on this, it is necessary to the discovery of diagnostic and therapeutic targets for breast cancer.

DnaJ Heat Shock Protein Family (Hsp40) Member B4 (DNAJB4), as a molecular chaperone, mainly regulates protein homeostasis. DNAJ proteins belong to the HSP40 family and can transfer substrates to Hsp70, stimulate its ATPase domain and confer specificity on this chaperone family [3]. DNAJB4 plays multiple roles in tumorigenesis, development, and maintenance in multiple cancer types. DNAJB4 was shown to inhibit melanoma invasion through diminished the expression of MMP2 and MMP9 [4]. DNAJB4 and Src SH3 domains interact to form carcinogenic complexes [5], which in turn affect the metastasis of lung cancer [6]. Low expression of DNAJB4 is correlated with poor prognosis of breast cancer [7]. It is unknown, however, exactly how DNAJB4 is involved in breast cancer.

Therefore, there is an urgent need to elucidate how DNAJB4 regulates breast cancer progression, providing a basis for in-depth study of breast cancer pathogenesis and the search for new therapeutic targets.

## Materials and methods

### Cell culture

Human breast cancer cell lines (MCF-7; BT-549; SKBR3; MDA-MB-231) and normal breast epithelial cell line (MCF-10A) were purchased from Procell (Wuhan, China). Cells were cultured in in DMEM containing 10% fetal bovine serum (FBS; C0400, VivaCell, Shanghai, China) and 1% penicillin/streptomycin (C0222, Beyotime, China) at 5% CO_2_ and 37 °C. The DNAJB4 overexpression lentivirus and negative control were purchased from GeneChem (Shanghai, China). The DNAJB4 overexpression lentivirus and negative control virus (MOI = 40) were transfected into MCF-7 cells using Lipofectamine 3000 (Invitrogen, USA) according to the manufacturer's protocol.

### Tissue specimens

Breast cancer tissue samples and adjacent tissues were obtained from the Department of Pathology, Affiliated Hospital of North Sichuan Medical College. The experimental protocols were approved by the Ethics Committee of the Affiliated Hospital of North Sichuan Medical College and all experiments were performed in accordance with the 1964 Helsinki Declaration and its later amendments (No. 2022ER308-1). The written informed consent was obtained from each patient.

### Immunohistochemistry

Paraffin-embedded breast cancer sections were placed overnight in an incubator (37℃) one day in advance. The next day, paraffin sections were placed in two boxes of xylene for 10 min each, followed by alcohol gradient treatment (100%, 100%, 95%, 90%, 85%, 80%) for 3 min each, and finally rinsed under running water for 5 min. Paraffin sections were boiled in boiled citrate for 15 min and rinsed with PBS after natural cooling. Next, 30% hydrogen peroxide drops were incubated 10 min, and then rinsed with PBS. Then, goat serum (1%) was added to the occlusion tissue (10–15 min). Serum was removed dropwise, and an appropriate amount of DNAJB4 antibody (1:100, 13064-1-AP, Proteintech), PD-L1 antibody (1:100, 14-5982-82, Invitrogen,), CD4 antibody (1:100, DF16080, Affinity, and CD8 antibody (1:100, AF5126, Affinity) was added at 4 °C overnight. Subsequently, the wet box was rewarmed at 37 °C for 15 min, and then rinsed with PBS. An HRP-conjugated secondary antibody was added and incubated 15–20 min, and then rinsed with PBS. DAB color development was performed for 1–5 min, tap water rinses for 5 min, hematoxylin counterstaining for 3 min, and hydrochloric acid alcohol differentiation for 1 s. Alcohol gradient treatment (80%, 85%, 90%, 95%, 100%, 100%) was performed for 3 min each. The resin seal was dried, and the samples were observed under a microscope. The staining intensity is rated as negative (0), weak (1), moderate (2), or strong (3). The staining extent is defined as the percentage of positive staining relative to the entire tumor area, with a score range of 0 (0%), 1 (1–25%), 2 (26–50%), 3 (51–75%), and 4 (76–100%). The total protein expression score is calculated by multiplying the intensity and positivity scores, with a total score range of 0–12 [8]. Image J was used to calculate the positive staining area on random regions of the tumor section.

### Western blot

RIPA lysis buffer was added to collect the cell supernatant, BCA reagent was used to determine the protein concentration, and SDS‒PAGE was used to isolate the total protein. The protein was transferred to the PVDF membrane and then blocked with 5% skim milk powder. Then, anti-DNAJB4 (1:2,000,13064-1-AP, Proteintech), anti-GAPDH (1:1000, ab9485, Abcam), anti-P-LATS1 (1:1000, 8654, CST), anti-LATS1 (1:1000, 3477, CST), anti-YAP (1:1000, 14074, CST), anti-P-YAP (1:1000, 13008,CST), anti-E-cadherin (1:1000, 14472, CST), anti-N-cadherin (1:1000, 13116, CST), and anti-Vimentin (1:1000, 46173, CST) were incubated overnight at 4 °C. The secondary antibodies were added and incubated 1 h at room temperature, and finally, membranes were detected using the enhanced chemiluminescence detection system.

### Quantitative real-time PCR (qRT-PCR)

Total RNA was extracted according to the kit instructions (RC112-01, Vazyme, China), and cDNA synthesis was performed using a reverse transcription kit (R323-01, MR101-01/02, Vazyme, China). With cDNA as a template, qRT-PCR was used to amplify the target genes DNAJB4, miR-183-5p, GAPDH, and U6. The relative expression level of the genes was calculated by the 2^−ΔΔCt^ method. DNAJB4 forward: CAGTTTGGGGAGGAAGGGTT, DNAJB4 reverse: TCGCCATGAAAGGTGTACCG, GAPDH forward: TGACTTCAACAGCGACACCCA, and GAPDH reverse: CACCCTGTTGCTGTAGCCAAA. miR-183-5p forward: 5'- CGCGTATGGCACTGGTAGAA -3', miR-183-5p reverse: 5'- AGTGCAGGGTCCGAGGTATT -3'. U6 forward: 5'- CTCGCTTCGGCAGCACA -3', U6 reverse: 5'- AACGCTTCACGAATTTGCGT -3').

### Cell proliferation assay

MCF-7 cells were cultured in 96-well plates (5,000 cells per well). After cell stabilization, Cell Counting Kit-8 reagent (CCK-8) (C0037, Beyotime, China) was added, and the absorbance at 450 nm was detected using a microplate reader at 0 h, 4 h, 24 h, 48 h, and 72 h to analyze the cell proliferation status. A total of 2000 cells per well were distributed in 6-well plates, fixed with paraformaldehyde for 10 min after 15 days, rinsed with PBS three times, and stained with crystal violet for 10 min. Subsequently, the cells were rinsed with PBS three times, the Petri dish was allowed to dry, and pictures were taken for statistical analysis.

### Cell scratch assay

Cells were prepared into suspension, 100 µl of suspension containing 30,000 cells was added in scratched insertion wells, and inserts were pulled out after cells had overgrown into the insertion wells. The cells were then cultured with serum-free medium, and the wound healing state was assessed at 6 h, 12 h, and 24 h. When the cells were confluent, imaging was stopped, and statistical analysis was performed.

### Luciferase assay

First, HEK293T cells were placed in 24-well plates, and then, hsa-miR-183-5p-targeted hsa-DNAJB4 DNA fragments (wild type and mutant) (0.8 µg) (GeneChem, China) and NC-mimics/miR-183-5p mimics (100 nM) (IBSBIO, China) were cotransfected for 48 h. The relative luciferase activity of the cells was assessed using Dual-luciferase Assay Kit (72050, Promega, USA) was used to detected the relative luciferase activity of the cells.

### Mouse xenograft assay

One hundred microliters of PBS containing 1 × 10^5^ 4T1 cells or 1 × 10^5^ DNAJB4-overexpressing cells were injected subcutaneously into the right axilla of female BALB/c mice (5 ~ 6 weeks). When the tumor diameter was 0.5 cm, a LATS-IN-1 (HY-138489, MCE, USA) suspension (10 mg/kg) was injected daily around the tumor for 7 days, and then, the mouse was sacrificed to remove the tumor (8 mice in each group). The tumor was embedded in paraffin block, and the tumor section was stained by immunohistochemistry to assess the expression of PD-L1 (1:100, 14-5982-82, Invitrogen, USA), CD4 (1:100, DF16080, Affinity, USA), and CD8 (1:100, AF5126, Affinity, USA). The animal experiment protocols were approved by the Animal Ethics Committee of North Sichuan Medical College (No.202203) and were performed in accordance with the National Institutes of Health Guide for the Care and Use of Laboratory Animals.

### Database analysis

Database analysis of DNAJB4 expression in tumors was performed using the TIMER2.0 database (http://timer.cistrome.org/). The prognostic effect of DNAJB4 on breast cancer patients was performed using the Kaplan‒Meier analysis (http://kmplot.com/analysis/). The gene expression RNA-Seq from 1226 patients with BRAC and 113 normal tissues were downloaded from TCGA database (https://portal.gdc.cancer.gov), and the data were normalized and log2 (x + 1) transformed. The target miRNA of DNAJB4 was predicted using the starBase database (https://starbase.sysu.edu.cn/) based on analysis of PITA, miRanda and TargetScan.

### Immune infiltration analysis

Using the R package “GSVA” to generate single-sample gene set enrichment analysis (ssGSEA), the relative tumor infiltration levels of immune cell types were quantified by integrating the gene expression levels [9]. The R package "Estimate" was used to calculate immune scores, stromal scores, and ESTIMATE scores [10]. The purity-correlated partial Spearman’s correlation was used to evaluate the correlations of DNAJB4 expression with immune infiltration based on TIMER database (https://cistrome.shinyapps.io/timer/).

### Statistical analysis

Statistical analyses were conducted using SPSS19.0 software and R version 3.6.3. Data are presented as mean ± standard error of the mean (SEM). Kolmogorov–Smirnov test was used to test normal distribution. Statistical differences among groups were quantified by paired two-tailed t tests, unpaired two-tailed t test or one-way analysis of variance (one-way ANOVA) followed by Tukey’s multiple comparison test. The KEGG enrichment analyses was visualized using the R packages “clusterProfiler” (version 3.15.3). A P value < 0.05 was considered as statistically significant.

## Results

### DNAJB4 expression is downregulated in breast cancer

**Fig. 1:**
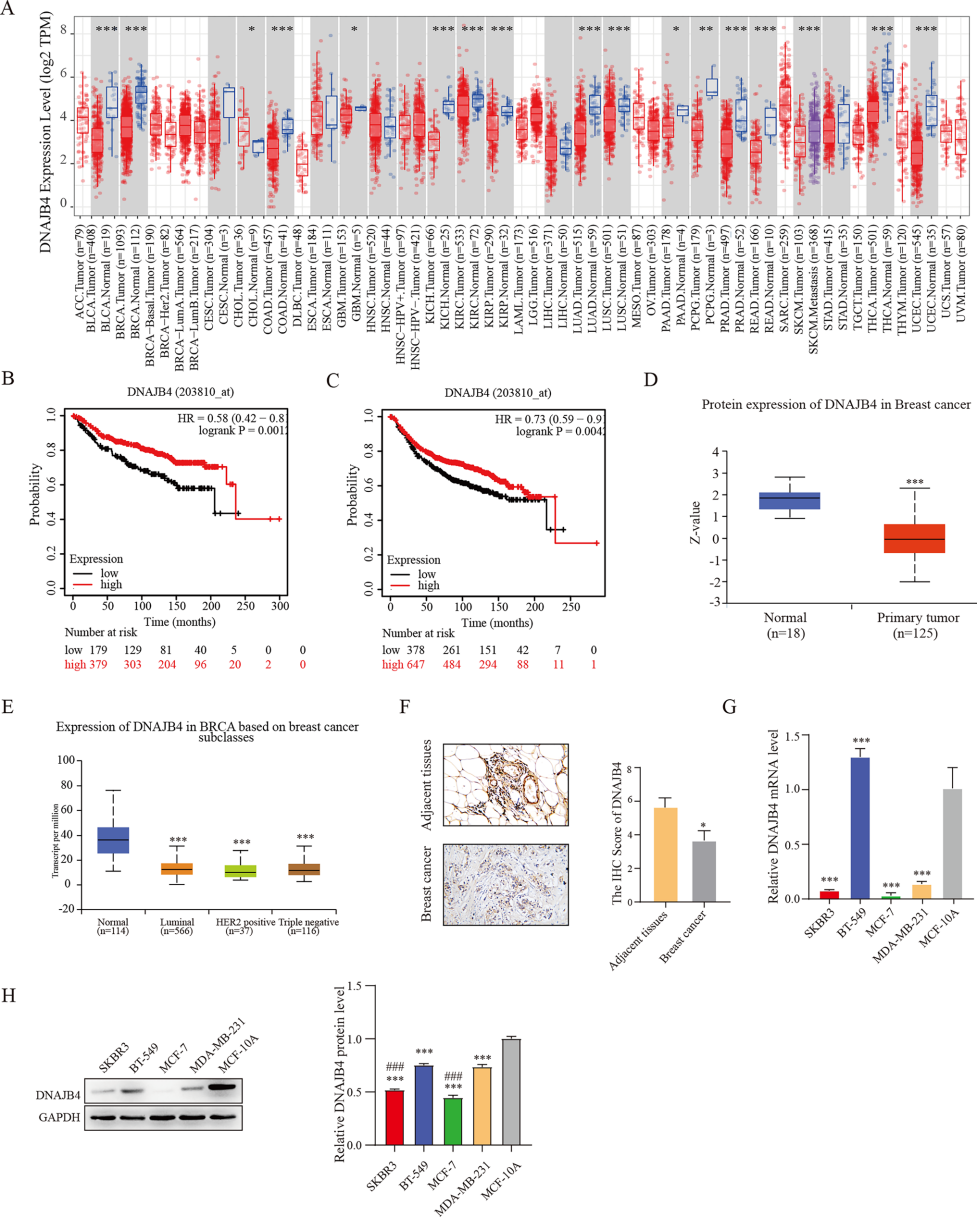
Expression of DNAJB4 in breast cancer. **A** The expression of DNAJB4 in breast cancer was assessed by using the TIMER2.0 database. **B-C** Survival curves of DMFS and RFS from the Kaplan‒Meier database. **D** The UALCAN database was used to assess DNAJB4 protein expression levels in breast cancer and** E** breast cancer subclasses. **F** Detection of DNAJB4 expression levels in human breast tissue by immunohistochemical chemical staining. **G**–**H** DNAJB4 expression in breast cancer cells was detected by qRT‒PCR and Western blots and. The data are expressed as means ± SEM of at least three independent experiments. * p < 0.05, *** p < 0.001, versus MCF-10A or adjacent tissue. ^###^ p < 0.001, versus BT-549 or MDA-MB-231

Through database analysis, we found a significantly downregulated DNAJB4 expression in breast cancer, bladder urothelial carcinoma, colon adenocarcinoma, kidney chromophobe and so on (Fig. 1A), and DNAJB4 protein level was also decreased in breast cancer tissues and different subclasses (Fig. 1D, E). Kaplan‒Meier analysis suggested that low DNAJB4 level was associated with worse distant metastasis-free survival (DMFS) and recurrence-free survival (RFS) in breast cancer patients (Fig. 1B, C). In addition, we performed immunohistochemical (IHC) staining to evaluate DNAJB4 expression in breast cancer tissue to verify the public dataset results. As shown in Fig. 1F, DNAJB4 expression was low in breast cancer samples. Moreover, normal breast cell line and breast cancer cell lines was used to assess the expression level of DNAJB4. DNAJB4 was significantly higher in MCF-10A cells and the lowest expression in the MCF-7 cells (Fig. 1G, H), so we chose the MCF-7 cells for subsequent experiments.

### DNAJB4 overexpression inhibits MCF-7 cell proliferation and migration

**Fig. 2:**
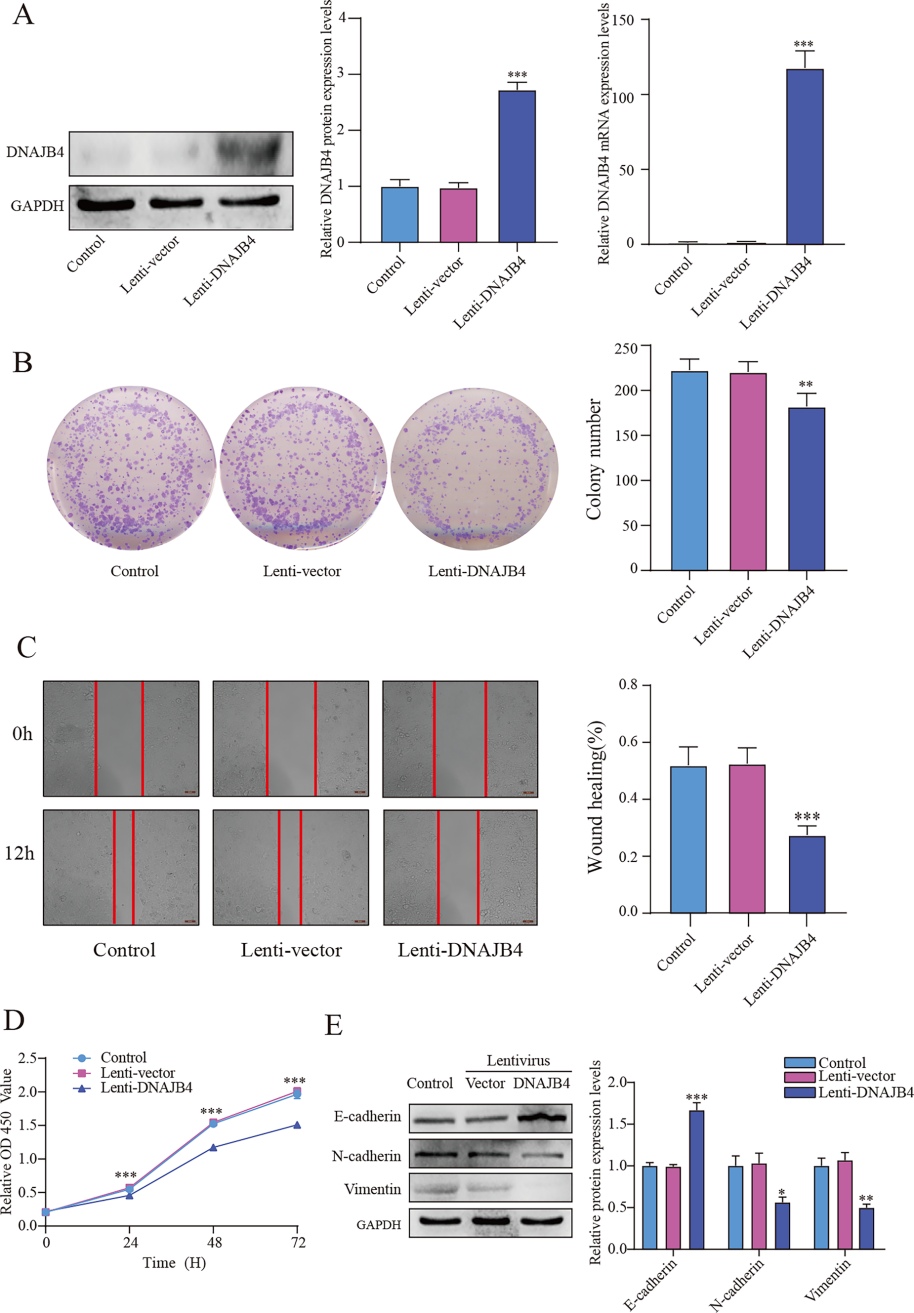
Overexpression of DNAJB4 inhibits the proliferation and migration of MCF-7 cells. **A** Validation of DNAJB4 overexpression efficiency by Western blots and qRT-PCR. DNAJB4 overexpression was followed by** B** colony formation assay, **C** scratch assays and** D** CCK-8 assays to assess the proliferation and migration changes of MCF-7 cells. **E** Overexpression of DNAJB4 followed by Western blotting to verify E-cadherin, N-cadherin and vimentin protein expression. The data are expressed as means ± SEM of at least three independent experiments. * p < 0.05, ** p < 0.01, *** p < 0.001, versus control

To evaluate the function of DNAJB4 in MCF-7 cells, we transfected the lentivirus to overexpress DNAJB4, and the efficiency of overexpression was confirmed by immunoblotting analyses and qRT‒PCR (Fig. 2A). The colony formation assays suggested that DNAJB4 overexpression significantly suppressed MCF-7 cells colony formation (Fig. 2B), and CCK-8 assay results further confirmed that the overexpression of DNAJB4 significantly suppressed MCF-7 cells proliferation (Fig. 2D). Scratch assay showed that compared with control group, the DNAJB4 overexpression inhibited MCF-7 cells migration (Fig. 2C). We further validated the influence of DNAJB4 overexpression on epithelial–mesenchymal transition (EMT). Compared with control group, DNAJB4 overexpression downregulated N-cadherin and vimentin expression and upregulated E-cadherin expression (Fig. 2E). Above results suggests that DNAJB4 may act as a tumor suppressor in MCF-7 cells.

### DNAJB4 overexpression activates the Hippo signaling pathway

**Fig. 3:**
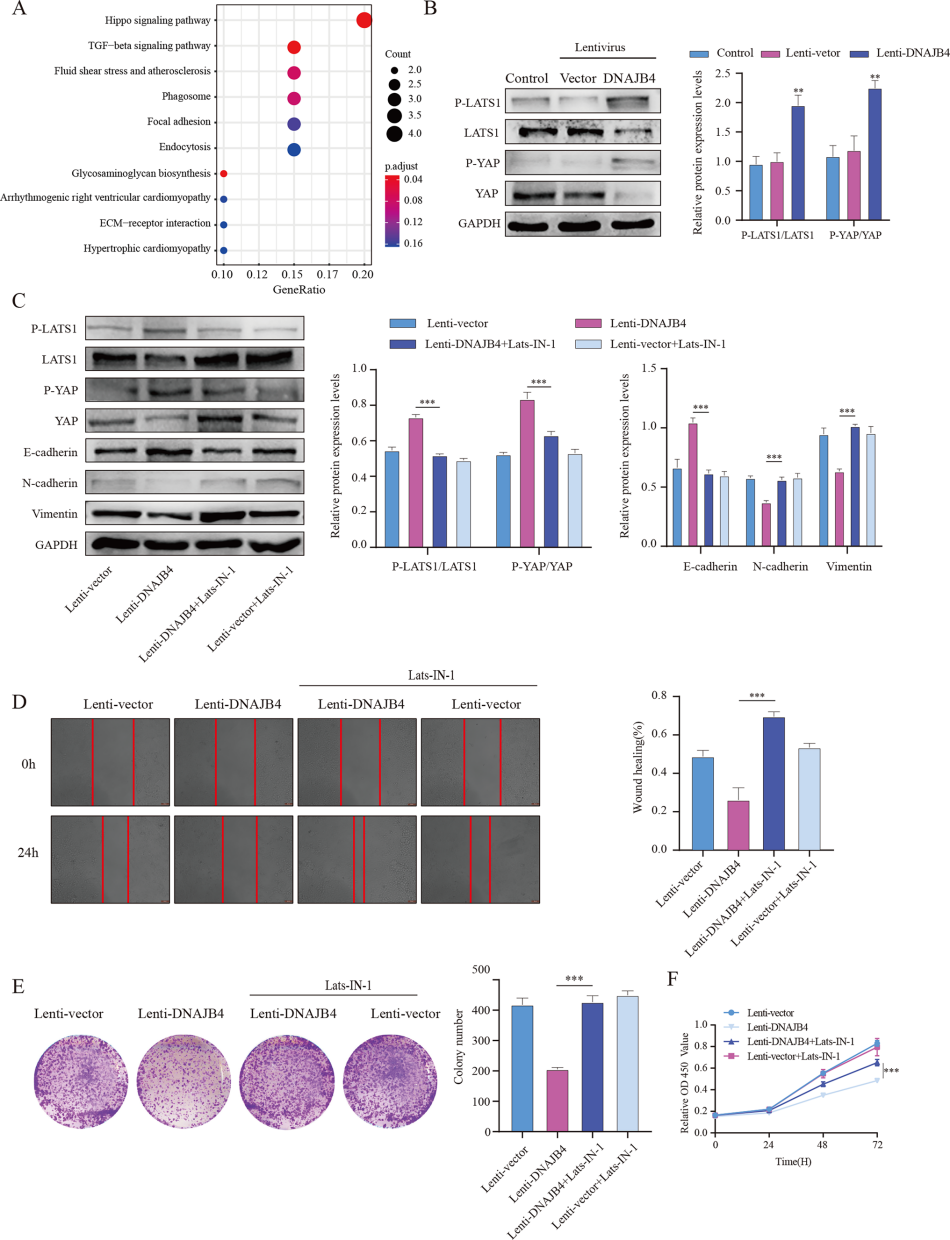
The DNAJB4-dependent Hippo pathway influences tumor behavior. **A** DNAJB4 was identified in the Hippo pathway through the TCGA-BRAC database. **B** After DNAJB4 overexpression, p-LATS1, p-YAP, LATS1 and YAP protein expression in MCF-7 cells was analyzed by Western blotting.** C** Western blot analysis detected E-cadherin, N-cadherin and vimentin protein expression after blocking the Hippo pathway by LATS-IN-1, while **D** scratch assays, **E** colony formation assay, and **F** CCK-8 assays detected the proliferation and migration of MCF-7 cells. The data are expressed as means ± SEM of at least three independent experiments. ** p < 0.01, *** p < 0.001, versus Lenti-control

To further explore the potential mechanism by which DNAJB4 regulates the biological behavior of MCF-7 cells, we identified the Hippo signaling pathway by using KEGG analysis based on the TCGA-BRAC dataset (Fig. 3A). Thus, to validate the role of the Hippo signaling in the DNAJB4 overexpression-effects against breast cancer progression, we investigated Hippo signaling in MCF-7 cells. DNAJB4 overexpression significantly increased the ratio of p-LATS1/LATS1 and p-YAP/YAP (Fig. 3B). Next, we used Lats1-IN-1, an ATP-competitive inhibitor of Lats kinases, to block the Hippo signaling pathway. The Lats1-IN-1 + DNAJB4 overexpression group showed significantly inhibited the expression of p-LATS1 and p-YAP (Fig. 3C,). Meanwhile, Lats1-IN-1 + DNAJB4 overexpression group significantly inhibited EMT (Fig. 3C). The proliferation and migration ability of MCF-7 cells was significantly restored after LATS-IN-1 inhibited the Hippo signaling (Fig. 3D–F). Together, these results indicated that DNAJB4 overexpression inhibits MCF-7 cells proliferation and migration by activating the Hippo signaling.

### MiR-183-5p negatively regulates DNAJB4

**Fig. 4:**
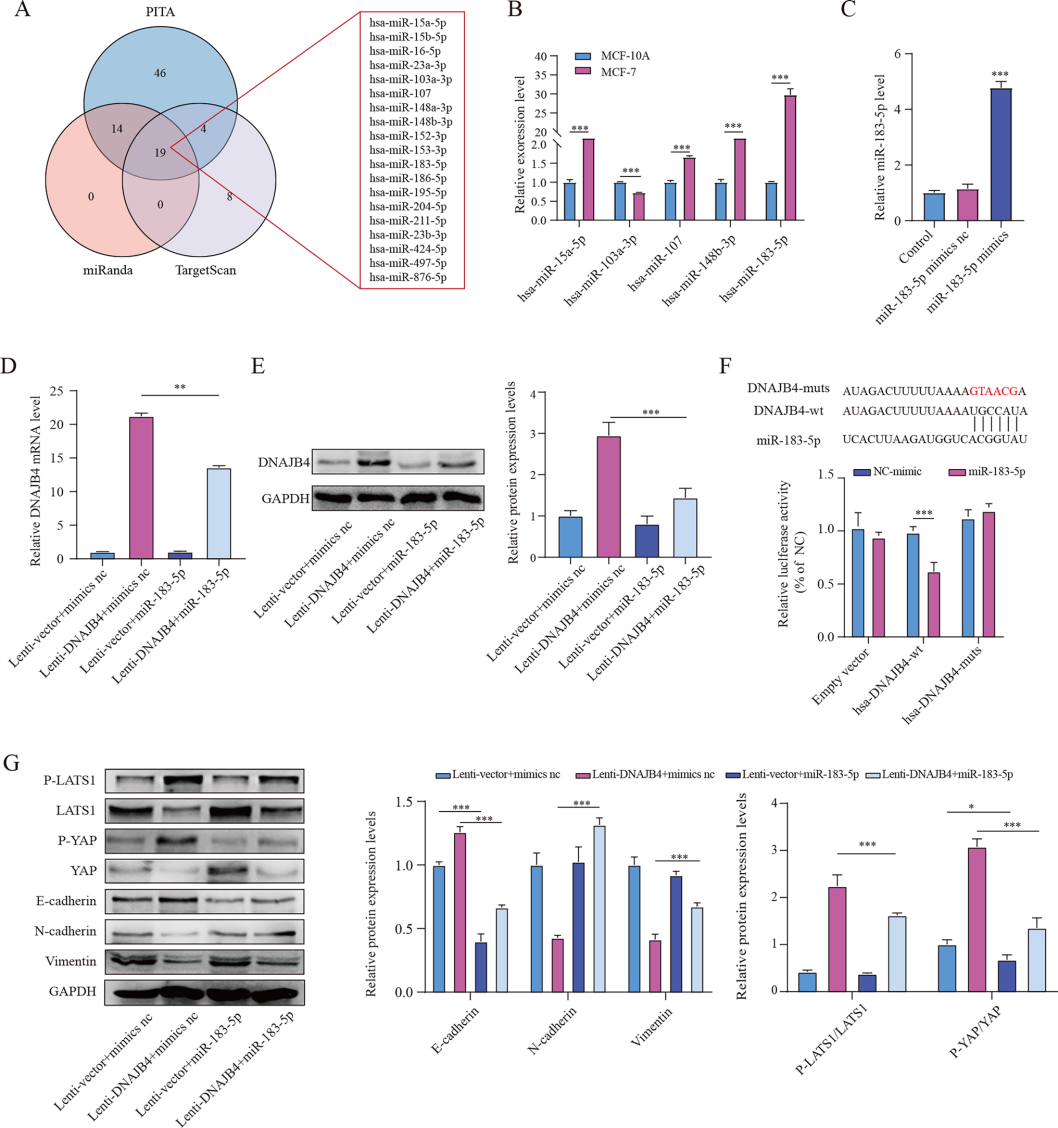
DNAJB4 is the target gene of miR-183-5p. **A** Prediction of miRNAs by the starBase database. **B** qRT-PCR was used to assess potential miRNAs expression levels. **C** miR-183-5p expression was detected by qRT-PCR in MCF-7 cells transfected with miR-183-5p. **D-E** The effect of miR-183-5p on DNAJB4 expression. **F** The binding site of miR-183-5p to the DNAJB4 mRNA 3ʹUTR was predicted, and luciferase activity was detected by a luciferase reporter gene. **G** The effect of miR-183-5p on the protein expression levels of p-LATS1, p-YAP, LATS1, YAP, E-cadherin, N-cadherin and vimentin. The data are expressed as means ± SEM of at least three independent experiments. * p < 0.05, ** p < 0.01, *** p < 0.001

MiRNAs regulates gene expression by binding 3′-UTRs of target genes. We used the PITA, TargetScan, and miRanda databases to identify putative miRNAs targeting DNAJB4. The database results suggested that five potential miRNAs were significantly negatively correlated with DNAJB4 (Fig. 4A). The miR-183-5p had the highest expression levels in MCF-7 cells (Fig. 4B). Subsequently, we transfected MCF-7 cells with miR-183-5p mimics or control (Fig. 4C). The results indicated that DNAJB4 expression was markedly reduced after transfected with mimics (Fig. 4D, E). DNAJB4 3ʹUTR contains a potential miR-183-5p binding sequence (Fig. 4F). To identify miR-183-5p direct inhibit DNAJB4 expression, we transfected wt DNAJB4 or mut DNAJB4 plasmid and mimics or control into HEK‐293T cells. The results suggested that miR-183-5p mimics obviously decreased the reporter activities of DNAJB4–3ʹUTR wt but did not change the reporter activities of DNAJB4–3ʹUTR mut (Fig. 4F). Nest, we assessed the influence of miR-183-5p on EMT and the Hippo signaling in MCF-7 cells. The results suggested miR-183-5p significantly promotes EMT and inhibited the Hippo signaling pathway, but DNAJB4 overexpression partially rescued the inhibition of EMT and the Hippo signaling pathway in MCF-7 cells (Fig. 4G). Thus, miR-183-5p could directly regulate DNAJB4 expression, thereby affecting Hippo signaling and changing tumor growth. Above results suggest miR-183-5p regulates EMT and the Hippo signaling by directly targeting DNAJB4 in MCF-7 cells.

### DNAJB4 overexpression inhibits xenograft tumor growth

**Fig. 5:**
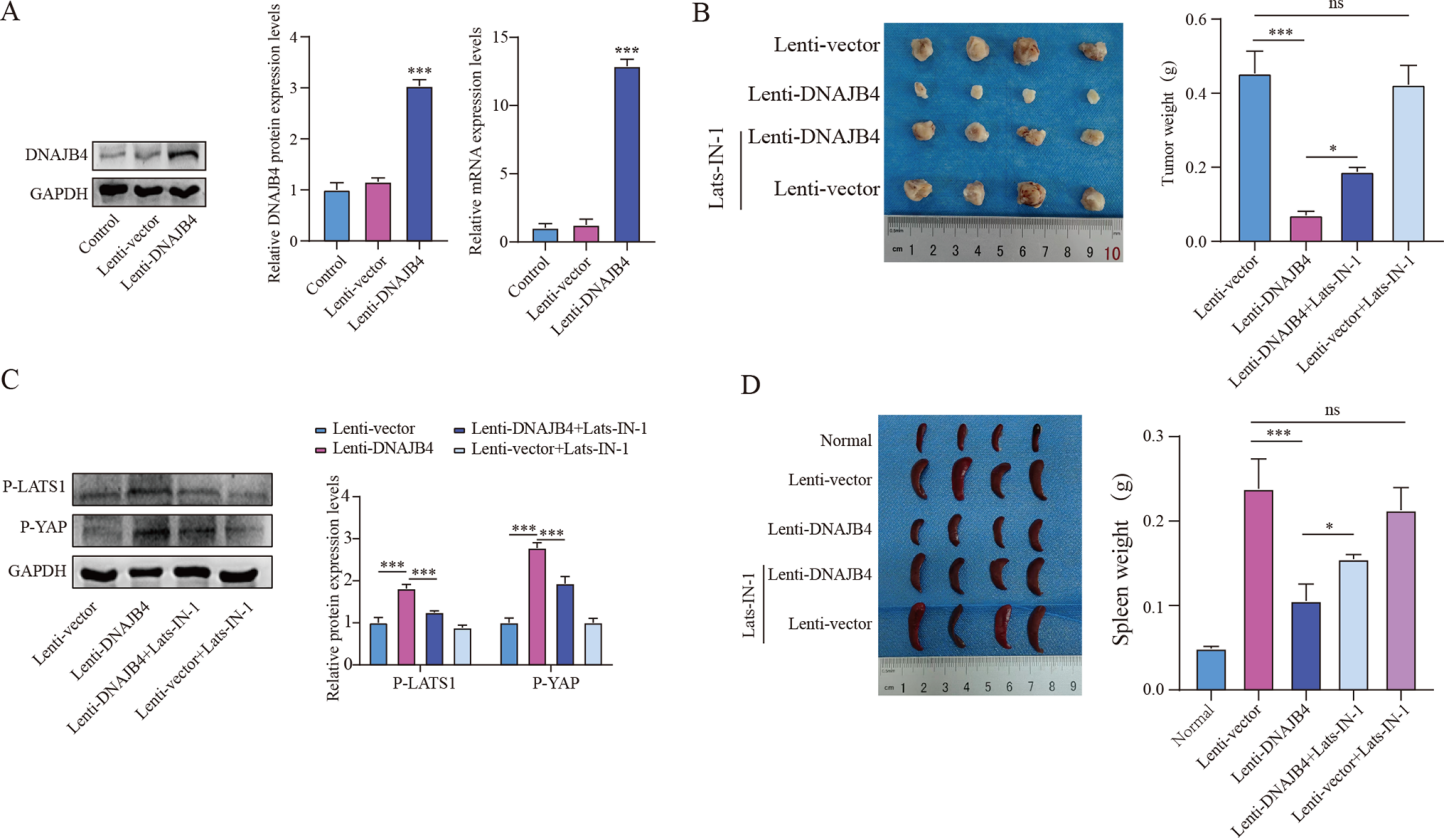
DNAJB4 overexpression suppresses tumor growth in vivo. **A** The overexpression efficiency of DNAJB4 in 4T1 cells was verified using qRT-PCR and Western blot (n = 3). **B** Images of the 4T1 breast cancer model tumor and weight statistics. **C** Western blot measurement of p-LATS1 and p-YAP protein expression in 4T1 breast cancer model tumors (n = 3). **D** Spleen images and weight statistics of the 4T1 breast cancer model. The data are expressed as means ± SEM. * p < 0.05, ** p < 0.01, *** p < 0.001

To validate the role of DNAJB4 in vivo, we established a xenograft tumor model with the mouse 4T1 cell in Balb/c mice. Mice were divided randomly into 4 groups (n = 8/group): Lenti-vector, Lenti-DNAJB4, Lenti-DNAJB4 + Lats-IN-1, and Lenti-vector + Lats-IN-1. The DNAJB4 overexpression 4T1 cell was constructed, and successful overexpression (Fig. 5A). Compared with the Lenti-vector group, Lenti-DNAJB4 significantly suppressed tumor size and weight, while combined treatment with Lats-IN-1 and Lenti-DNAJB4 significantly increased tumor size and weight (Fig. 5B). Western blot revealed a noticeable increased in the expression of p-LATS1 and p-YAP in Lenti-DNAJB4 group. However, combined treatment with Lats-IN-1 and Lenti-DNAJB4 inhibited the phosphorylation of LATS1 and YAP (Fig. 5C). These data suggest that targeting DNAJB4 suppresses xenograft breast tumor growth by activating the Hippo signaling pathway. Interestingly, we found that spleen weight was lower in the Lenti-DNAJB4 group, while the spleen weight of the Lenti-DNAJB4 group after Lats-IN-1 treatment was significantly increased relative to that of the Lenti-DNAJB4 group (Fig. 5D). Above results suggest, DNAJB4 overexpression inhibits xenograft tumor growth.

### DNAJB4 expression is associated with immune infiltration

**Fig. 6:**
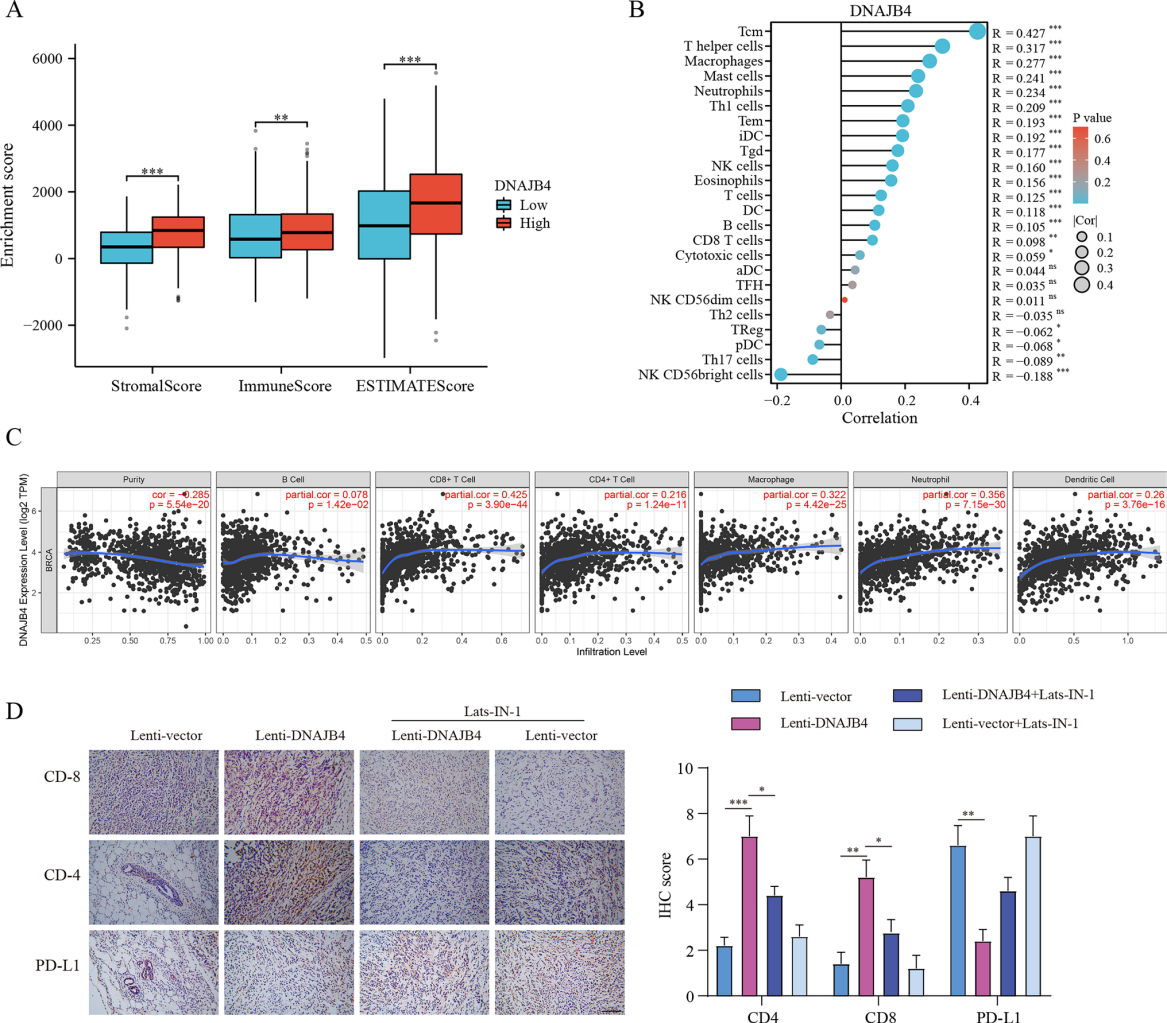
Correlation of DNAJB4 expression with immune infiltration level. **A** DNAJB4 expression is correlated with ImmuneScore, StromalScore and ESTIMATEScore. **B** DNAJB4 expression is correlated with the infiltration of immune cells in BRAC using the ssGSEA.** C** DNAJB4 expression is correlated with immune infiltrations in BRAC using the TIMER database. **D** Immunohistochemical staining and quantification of CD4, CD8, and PD-L1 expression in tumor tissues of the 4T1 breast cancer model (n = 5), Scale bar = 200 μm. The data are expressed as means ± SEM. * p < 0.05, ** p < 0.01, *** p < 0.001

Multiple lines of evidence suggest that aside from regulating tumor progression, the Hippo pathway also modulates tumor microenvironment. We hypothesized that DNAJB4 therapy may also influence tumor immunity. As shown in Fig. 6A, high expression of DNAJB4 was associated with the increasement of stromal, immune scores, and ESTIMATE scores. The ssGSEA results showed that DNAJB4 expression was positively correlated with infiltration of Tcm, T helper, macrophages, mast cells, neutrophils, and Th1 cells. It was negatively correlated with infiltration of NK CD56 bright cells, Th17 cells, pDC, and Treg cells (Fig. 6B). In addition, DNAJB4 expression was positively correlated with infiltration of B cells, CD4 + T cells, CD8 + T cells, macrophages, neutrophils, and dendritic cells (Fig. 6C). As shown in Fig. 6D, in vivo experimental results suggested that Lenti-DNAJB4 could increase the infiltration of both CD4 + and CD8 + T cells, and suppress PD-L1 expression compared to that of the Lenti-control group. Thus, these results indicate that DNAJB4 may serve as a major regulator of breast cancer progression.

## Discussion

**Fig. 7:**
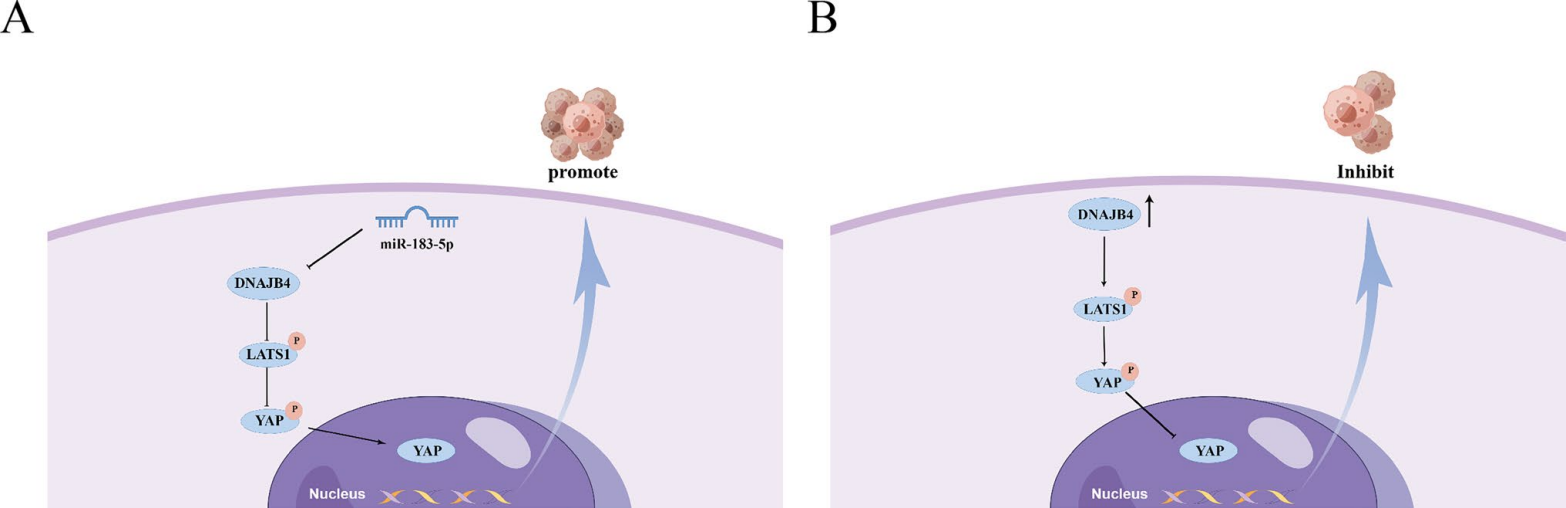
The proposed working model of miR-183-5p relies on DNAJB4 to regulate the Hippo pathway in breast cancer

Although some breakthroughs has been made [7], the incidence and mortality of breast cancer continue to increase, and the age trend of patients is getting younger [11]. Hence, it is necessary to explore the mechanisms underlying breast cancer. The current study found that DNAJB4 is downregulated in a variety of tumors. However, some genes have different molecular mechanisms in different tumor cells, leading to significant differences in their expression levels. For instance, DNAJB4 is upregulated in SKCM metastasis, and its loss can inhibit EMT and reduce lung cancer metastasis [5]. Consistent with previous studies, the current results indicated that DNAJB expression were significantly higher in BT549 cell than in other types of breast cancer cell lines. In addition, some studies have revealed that interference with DNAJB4 expression could enhance the biologic behavior of basal-like MDA-MB-231 cell lines [7]. However, it is not clear how DNAJB231 contributes to breast cancer progression. Our results found that DNAJB4 expression was downregulated in breast cancer tissues and lower in luminal-like cell lines MCF7 than basal-like MDA-MB-231 cell, and DNAJB4 overexpression enhanced the proliferation and migration ability of MCF-7. The database found that when DNAJB4 expression was low, breast cancer patients had a poor prognosis. Therefore, we suggest that DNAJB4 may play an indispensable role in breast cancer (Fig. 7).

Hippo pathway can control organ size and maintain tissue stability by regulating cell proliferation and apoptosis [12, 13]. Hippo pathway main consists of MST1, LATS1 and Mob1, all of which are activated by phosphorylation. The LATS1 and MST1 were identified as tumor suppressor genes while regulating YAP phosphorylation [14–16]. Moreover, YAP and TAZ as key factors of Hippo pathway, which exert their oncogenic functions by binding to TEAD [17, 18]. LATS1, Mob1, and YAP were shown to be deficient in hepatocellular carcinoma, and the phosphorylation of LATS1, Mob1, and YAP could activate the Hippo pathway and thereby suppressed tumors [19]. It also is related to breast cancer, and studies have shown that YAP downregulation inhibits lung metastasis in mouse models of breast cancer [20]. Decreased expression of LATS1 or LATS2 leads to tumor enlargement and lymph node metastasis [21, 22]. In addition, inhibition of the Hippo pathway could enhance cell proliferation and EMT [23, 24]. Here, we discovered that DNAJB4 overexpression could suppress migration and proliferation ability of MCF-7 cell, and the increased expression of DNAJB4 inhibited the progression of EMT. In addition, we found the ratio of p-LAST/LATS1 and p-YAP/YAP was significantly increased, which indicated that the increased expression of DNAJB4 prompted the activation of the Hippo pathway. We investigated whether the DNAJB4-dependent Hippo pathway regulated tumors progression. Using Lats-IN-1 to block Hippo, we revealed that the proliferation and migration of MCF-7 cells were significantly increased in the Lenti-DNAJB4 + Lats-IN-1 group compared with the Lenti-DNAJB4 group. Interestingly, overexpression of DNAJB4 + blocking of the Hippo pathway contributed to a higher would healing area compared to the control. This may be because the Lats-IN-1β inhibits Hippo pathway through additional potential mechanisms. However, the limitation of this study is that only one cell line was used in the rescue experiments and in animals’ studies, and our results need to be further confirmed in future studies.

The reason for the low expression of DNAJB4 in breast cancer is still unclear, but existing evidence suggests that microRNAs have regulatory effects on many genes. The PITA, TargetScan, and miRanda databases were used to predict potential miRNAs that regulates DNAJB4 expression. Previous studies have also found that miR-183-5p promotes hepatocellular carcinoma progression via regulate IRS1 expression [25] and promotes the invasion and proliferation thyroid cancer cells by regulating FOXO1 [26]. In addition, we also found that miR-183-5p mimics could reverse the activation of the Hippo pathway caused by DNAJB4 overexpression. Furthermore, Hippo pathway regulates tumor immune microenvironment and have a crucial role in tumor immunotherapy [27, 28]. In this study, we found a correlation between the expression of DNAJB4 and immune infiltration. The high expression of DNAJB4 is related to high levels of stromal cell infiltration and lower tumor cell purity in BRAC [10]. The ssGSEA results showed that the expression of DNAJB4 was significantly positively correlated with immune infiltration of Tcm, T helper cells, Macrophages, and Mast cells, and negatively correlated with NK CD56 bright cells and Th17 cells. The current study found that LATS1/2 deficiency enhanced tumor immunogenicity, leading to tumor destruction through an enhanced antitumor immune response [28]. Similarly, in vivo experiments verified that DNAJB4 influenced tumor growth through Hippo regulation of the tumor immune microenvironment.

## Conclusions

In summary, DNAJB4 is regulated by miR-183-5p, which affects the activation of the Hippo signaling and thus alters the tumor immune microenvironment and ultimately regulates the biological behavior of MCF-7 cells. Our findings indicates that DNAJB4 may be a new target for breast cancer therapy.

## Data Availability

The datasets generated and/or analyzed during the current study are available from the corresponding author on reasonable request.
